# Automated Quality Control for Sensor Based Symptom Measurement Performed Outside the Lab

**DOI:** 10.3390/s18041215

**Published:** 2018-04-16

**Authors:** Reham Badawy, Yordan P. Raykov, Luc J. W. Evers, Bastiaan R. Bloem, Marjan J. Faber, Andong Zhan, Kasper Claes, Max A. Little

**Affiliations:** 1 School of Engineering and Applied Sciences, Aston University, Birmingham B4 7ET, UK; yordan.raykov@gmail.com (Y.P.R.); maxl@mit.edu (M.A.L.); 2 Institute for Computing and Information Sciences, Radboud University, 6525 EC Nijmegen, The Netherlands; Luc.Evers@radboudumc.nl; 3 Department of Neurology, Donders Institute for Brain, Cognition and Behavior, Radboud University Medical Center, 6525 HR Nijmegen, The Netherlands; Bas.Bloem@radboudumc.nl; 4 Radboud Institute for Health Sciences, Scientific Center for Quality of Healthcare, Radboud University Medical Center, 6525 EZ Nijmegen, The Netherlands; Marjan.Faber@radboudumc.nl; 5 Department of Computer Science, Johns Hopkins University, Baltimore, MD 21218, USA; azhan2@jhu.edu; 6 UCB Biopharma, B–1070 Brussels, Belgium; Kasper.Claes@ucb.com; 7 Media Lab, Massachusetts Institute of Technology, Cambridge, MA 02139, USA

**Keywords:** Bayesian nonparametrics, clinimetric tests, Parkinson’s disease, pattern recognition, quality control, remote monitoring, segmentation, wearable sensors

## Abstract

The use of wearable sensing technology for objective, non-invasive and remote clinimetric testing of symptoms has considerable potential. However, the accuracy achievable with such technology is highly reliant on separating the useful from irrelevant sensor data. Monitoring patient symptoms using digital sensors outside of controlled, clinical lab settings creates a variety of practical challenges, such as recording unexpected user behaviors. These behaviors often violate the assumptions of clinimetric testing protocols, where these protocols are designed to probe for specific symptoms. Such violations are frequent outside the lab and affect the accuracy of the subsequent data analysis and scientific conclusions. To address these problems, we report on a unified algorithmic framework for automated sensor data quality control, which can identify those parts of the sensor data that are sufficiently reliable for further analysis. Combining both parametric and nonparametric signal processing and machine learning techniques, we demonstrate that across 100 subjects and 300 clinimetric tests from three different types of behavioral clinimetric protocols, the system shows an average segmentation accuracy of around 90%. By extracting reliable sensor data, it is possible to strip the data of confounding factors in the environment that may threaten reproducibility and replicability.

## 1. Introduction

In recent years, sensors embedded in smartphones and wearable devices have become ubiquitous and have evolved to the point where they can be used in areas such as healthcare [1,2], environmental monitoring [3,4] and transport [5]. In healthcare, for example, smartphone sensors have been successful at detecting the symptoms of neurological disorders such as Parkinson’s disease (PD) [6]. Through a smartphone application, on-board sensors in the smartphone capture the behavior of the user while they carry out a simple clinimetric (the science of measuring symptoms through quantitative instruments) test protocol, such as walking in a straight line with the smartphone in their pocket [7], to detect the key symptoms of the disease. Collecting objective symptom measurements with clinimetric testing performed on technologies such as smartphones [8,9] or portable and wearable devices such as smartwatches [10] and wearable IMUs [11,12] eliminates much of the subjective bias of clinical expert symptom measurement, while also allowing for remote, long-term monitoring of patient health [13]; in contrast to the current “snapshot” in time obtained during a clinical visit. Thus, remote, long-term monitoring allows for improved analysis of a patient’s health and outcomes. Usually, data from such sensors are collected and analysed under a set of clinimetric test protocol assumptions, such as the type of behavior to be carried out to probe for specific symptoms. Getting the assumptions of a test protocol to hold outside controlled lab settings is a universal problem, since uncontrollable confounding factors in the environment, such as unexpected behaviors, can have an adverse impact on the measurement process.

Analysing confounded or contaminated data produces misleading, biased results, which are inherently non-reproducible and non-replicable [14]. In many consumer applications that use sensor technologies, such data collection quality issues may not be that important since the consequences are not dire, but they are of critical importance in the medical sciences. Non-reproducible results in clinimetric studies could have significant implications for an individual’s health, as they may provide misleading information that may be acted upon.

In the case of fixed position (non-wearable) sensors, application-specific algorithms for quality control that exploit variation patterns in the sensor measurements have recently been proposed [15]. However, when it comes to quality control of sensors used for patient monitoring, the user behavior is the main source of variation in the sensor measurements; thus, a novel and more general framework that can adequately model human behavior is needed.

The quality control process can be viewed as the problem of locating different user behaviors and assessing if these behaviors are in adherence with the protocol assumptions. Yet, it is not feasible to approach this problem using methods used for “activity recognition”. Typically, in activity recognition in “ubiquitous computing” applications, the sensor data are segmented into windows of fixed alignment and equal duration, and then, a hand-crafted set of features is extracted from each window [16]. Subsequently, these features are used to train a classification algorithm that predicts the activity in each window. One of the problems is that both the hand-engineering of features and the training of the classifier in such systems depends heavily on having detailed, labeled information about which activities actually occurred. However, outside the lab, such information is rarely available. The second problem with current approaches to activity recognition, is that usually, they rely on modeling frequency domain features, which lose a lot of their predictive power when estimated directly from windows of non-stationary data [17].

The performance of heavily “fine-tuned” machine learning systems for activity recognition are misleading if the sensor data collected outside the lab are drawn from a different distribution to that collected in the lab and used for training the system [18]. This problem is compounded when using high performance nonlinear classification algorithms (such as convolutional neural networks [19], random forest classifiers [20] or support vector machines [21]) on a large number of features all estimated from a training distribution of questionable relevance in practice. This is because complex classification algorithms are more sensitive to the idiosyncrasies of the data distribution [22].

To address these issues, in this study, we propose a unified algorithm framework for automated assessment of clinimetric sensor data quality, i.e., the extent to which the data adhere to the assumptions of the clinimetric test protocols. Combining both parametric and nonparametric signal processing and machine learning techniques, we demonstrate the scope, effectiveness and interpretability of this framework by applying it to multiple sensor types and clinimetric tests for assessment of PD. PD is a brain disease that significantly affects voluntary movement [23]. Symptoms of PD include slowness of movement (bradykinesia), trembling of the hands and legs (tremor), absence of movement and loss of balance (postural instability). Across 100 subjects and 300 clinimetric tests from three different types of behavioral clinimetric protocols, the system shows an average segmentation accuracy of around 90% when compared to a human expert performing the same quality control task manually. We focus on data collected from smartphone sensors deployed outside the lab, as these are the most ubiquitous devices available for objective symptom measurement in practice.

### 1.1. Related Work

Smartphones and wearables are being increasingly recognised as potential tools for remote monitoring, diagnosis and symptom assessment of patients with various conditions. Recent healthcare applications of such technologies can be found in wider reviews such as Ozdalga et al. [24], Mosa et al. [25] and Kubota et al. [22]. For such devices to become useful clinical tools, they have to be deployed in natural environments, such as in the home or office. However, data collected from such devices outside the lab are confounded by many irrelevant factors related to the occupied environment or the individual’s behavior. Moreover, we often do not have the ground truth of the user’s behavior during remote monitoring. This severely challenges our ability to translate our findings in the lab to more realistic scenarios such as remote monitoring in users’ homes. One can view this issue as a quality control problem in which confounding factors need to be removed from data collected outside the lab.

Assuming data collected outside the lab is correctly and carefully labeled, the quality control process can often be done manually by inspecting the labels and removing any behaviors that violate clinimetric test protocols. However, in practice, this is rarely feasible since we have very little labeled information about the user outside the lab. Data collected outside the lab are often labeled to provide further context in data analysis, including manual hand labeling by a trained expert, user self-annotations [26] and video monitoring of users. Video recordings are subsequently manually annotated and are common in continuous patient monitoring systems including tremor detection [27] or PD disease severity assessment [28]. Using video annotations significantly complicates the experimental setup. This is why often, only certain parts of the video recording are observed to obtain labels for parts of the data collected, since going through hours of video footage can be time consuming and costly. Alternatively, self-annotations or self-report diaries usually deviate significantly from expert assessment, at least for neurological disorders such as PD [29]. Manual expert annotation of sensor data is also not always objective and can typically provide only broad indications of user behavior or health status. Unfortunately, these issues are often overlooked when it comes to the evaluation of clinimetric testing tools tested in the lab [30,31,32]. Looking outside of the domain of remote health monitoring, the quality control of sensor data has been broadly approached by: applying some hard-coded constraints for identifying simpler data anomalies or developing more adaptive data-driven approaches.

1. Pre-specified fixed constraints often appear in the form of: high/low-pass filters; sensor type and sensor location selection procedures; anomaly detection, etc. For example, changes in the orientation of a device introduce unwanted interruptions in sensor data such as those collected from an accelerometer during monitoring (for more information, see Section 2.3.1). This problem is usually approached by applying a high-pass filter to the data with a pre-specified threshold of 0.5 Hz [28]. The threshold value is not learned from the data, but is fixed empirically based on domain knowledge. Although this approach is over-simplifying assumptions about the data, such methods have been shown to be very useful for tackling problems related to hardware and easy to define events.
2. In contrast, data-driven quality control techniques look at the structural differences in the data and aim to learn some relation between data that represent the two classes (i.e., good quality vs. bad quality data). For example, hidden Markov models (HMMs) and dynamic Bayesian networks [33,34] have been used in multiple domains to detect anomalous sensor readings and in describing uncertainty associated with sensor readings [33], such as those collected from temperature and conductivity sensors. However, data-driven approaches have not been used before to address more sophisticated experimental setups, which is the case in remote patient monitoring applications.

In the problem of remote patient monitoring, the most challenging part of quality control comes down to modeling human behavior and detecting activities that violate clinimetric test protocols. Activity classification frameworks have been used for the detection and assessment of specific PD symptoms [35,36,37]. For example, Zwartjes et al. [35] and Salarian et al. [36] developed an in-home monitoring system that detects specific behaviors and subsequently predicts the movement impairment severity of these activities in terms of common PD symptoms. Both studies use existing activity classification methods that rely on training on a pre-defined, specific set of motion-related features, which can be used to distinguish between a selected, fixed set of activities. The problem is that in general, it is not feasible to anticipate the entire behavioral repertoire of a participant during any clinimetric test conducted outside the lab. Existing activity recognition systems require a rich set of features to be extracted from the input data, and the choice of features depends on the activities between which we wish to discriminate [16]. For these reasons, most traditional supervised activity recognition systems are not feasible for quality control in the context discussed in this paper, where any set of a potentially infinite range of behaviors could be encountered.

An alternative solution is to segment data into variably-sized windows in an unsupervised way, i.e., where the specific activity in each segment is not specified [38]. For example, HMM-based techniques have been studied for both supervised, as well as unsupervised segmentation of activities; however, often, activity-dependent features still need to be identified and extracted to achieve satisfying results. Gupta and Dallas [39], Bhattacharya et al. [40], Guo et al. [41] and others have proposed more adaptive segmentation approaches that do not rely on a specified a priori fixed set of features, but use feature selection tools to choose the most appropriate features for the segmentation task at hand. For example, in Guo et al. [41], a rich set of features is extracted from fixed width (5–10 s) windows of multi-dimensional sensor data, and principal component analysis (PCA) is then used to project the feature space into a lower dimensional space where activities can be segmented based on the lower dimensional representation. This is mostly done through the use of unsupervised bottom-up hierarchical clustering. As the user’s activities change, the PCA in Guo et al. [41] is recomputed and chooses a different projection depending on the monitored tasks. In this work, we propose a data-driven quality control approach that also uses unsupervised segmentation to group and locate different user activities. However, by using more advanced probabilistic modeling approaches, we bypass the need for windowing the raw data, which allows one to detect very short-term (less than 0.05 s) test protocol violations and also almost bypasses any need for sensor-specific feature engineering.

### 1.2. Overview

**Figure 1:**
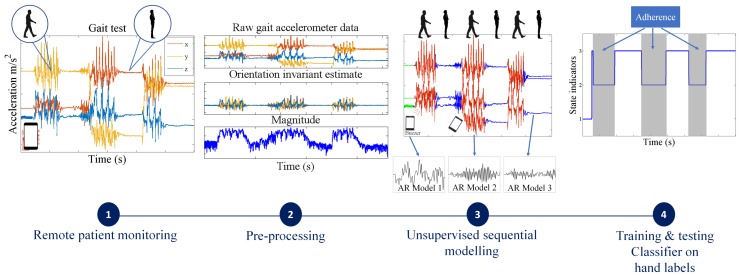
Overview of the proposed algorithmic framework for data quality control of sensor data for behavioral clinimetric testing. The first stage (starting from the left) involves collecting sensor data outside the lab. The second stage consists of removing confounding factors from the data such as the effect of the orientation of the device. The third stage involves unsupervised segmentation of the data into intervals in which the user performs similar activities. In the final stage, a simple, interpretable classifier is trained to predict which intervals are associated with adherence to, and which with violations of, the test protocols.

In this section, we describe the stages of our proposed unified framework for quality control of clinimetric test sensor data (Figure 1). As an example application, we apply the system to multiple clinimetric smartphone tests for PD symptom monitoring. We describe the test protocols for these clinimetric tests in Section 2.

After data collection of the smartphone tests, we apply practical preprocessing steps, which depending on the type of sensor, produce a more discriminative representation of the data without discarding any essential structure. For example, in the case of accelerometer data from sensors embedded in smartphones, we can remove the effect of orientation changes of the smartphone. This is because device orientation is usually a confounding factor in clinimetric testing. For high sample-rate voice data, we segment the original signal into short-duration, 10-ms windows and extract features such as the energy or spectral power in each window, instead of modeling the raw data directly. Unlike most of the existing machine learning strategies for processing sensor data, we do not rely on a large number of features extracted from each sensor type; we apply minimal transformations to the raw data keeping them at a relatively high sampling frequency and directly fit a flexible probabilistic model aiming to capture the structure of importance in the quality control problem at hand.

Once the sensor data have been preprocessed accordingly, we fit a segmentation model to each of the sensor signals directly in an unsupervised manner, and split the data into segments of varying duration. Depending on the complexity of the data produced by different clinimetric tests, we propose two different segmentation models that vary in terms of flexibility and computational simplicity:

1. For simpler quality control problems, we develop a Gaussian mixture model (GMM)-based approach, which attempts to cluster the raw signal into two classes: data adhering or violating the test protocol. Since GMMs ignore the sequential nature of the sensor data, we pass the estimated class indicators through a running median filter to smooth out unrealistic frequent switching between the two classes.
2. We also propose a more general solution that involves fitting flexible nonparametric switching autoregressive (AR) models to each of the preprocessed sensor signals. The switching AR model segments the data in an unsupervised manner into random (unknown) numbers of behavioral patterns that are frequently encountered in the data. An additional classifier is then trained to discriminate which of the resulting variable-length segments represent adherence or violation of the test protocol. We demonstrate that a simple multinomial naive Bayes classifier can be trained using a strictly limited amount of labeled data annotated by a human expert. Since the instructions in any clinimetric test protocol are limited, whereas the number of potential behavioral violations of the protocol are not, we assume that any previously unseen segments that we detect are a new type of violation of the specified instructions of the protocol.

We have detailed both methods for automated quality control in Section 2.4.

## 2. Methodology

### 2.1. Data Collection

To illustrate our novel framework in practice, we use data from the Smartphone-PD study [6], which utilises an Android OS smartphone application to capture raw sensor data from the digital sensors embedded in the device. Smartphones come equipped with a wide variety of sensors including an accelerometer, microphone, gyroscope, magnetometers and barometer. Subjects were enrolled worldwide through an email database and sent an email with an installation link that automatically installs the application directly onto the subjects’ personal smartphones. The application prompts the user to undertake short (less than 30 s) self-administrated clinimetric tests designed to elicit the symptoms of PD. These tests are: (1) voice test (microphone), which measures impairment in the production of vocal sounds; (2) balance test (accelerometer), which measures balance impairment (postural instability); and (3) walk test (accelerometer), which measures impairment in a user’s walking pattern (see Table S1, Supplementary Material). Subjects were asked to conduct these tests at home, consecutively, twice per day.

### 2.2. Hand-Labeling for Algorithm Evaluation

In order to evaluate the performance of the automated quality control algorithms developed here, some reference data are needed. To this end, the smartphone accelerometer tests were visually inspected, and the voice tests were played back to hand-label sections of the data that represent behaviors that adhere to the test protocol or violate it (those labels will later appear as $u_{1},\ldots ,u_{T}$, which takes the value of one for adherence and the value of two for violation). Note that this is an inherently subjective process, and we cannot be sure of the exact activity occurring during any period of time. It is important to point out that we do not aim to identify specific activities (such as climbing stairs, sitting, standing, etc.) in the data as this is implausible, since the number of activities that can occur outside the lab is effectively infinite. Instead, we only aim to segment the data associated with different behaviors. This segmentation approach is less dependent on labeled data, and hence, the approach is general enough to be applied to most clinimetric sensor-based tests. To better understand the ability of our model to segment the data into different behaviors, we conduct our own controlled experimental smartphone tests in which we have the “ground truth”, and report our results in Table 1. In contrast, the “ground truth” labels for the smartphone data collected remotely are not available; thus, we make use of hand-labels provided by a domain expert, i.e., adherence vs. non-adherence for each segment. This type of labeling is always unavoidably somewhat subjective; thus, our aim is not to create an algorithm that blindly reproduces the hand-labels. Instead, we aim to develop an approach that learns the major structural differences between data adhering to, and data violating, test protocols in a mostly unsupervised way. Our ability to identify structural differences in the data depends to a certain extent on having data to train the model in order to extract the structure relevant for determining the parts of the data where the protocol has been followed. This synthesis of a probabilistic model with human expertise means that neither have to be perfect, and yet, both complement each other to produce a more useful and reliable discrimination. We are also careful to specify a hand-labeling protocol so that this labeling can be reproduced (see Table S1, Supplementary Material).

We labeled data from 100 subjects (voice, balance and walking tests) from the Smartphone-PD data, randomly selecting 50 PD patients (25 males and 25 females) and 50 healthy controls (25 males and 25 females). Subjects are age and gender-matched (two-sample Kolmogorov–Smirnov test) to rule out potential age or gender confounders.

The demographics of the 50 PD subjects used in this study are as follows: Ages range from 30 s to 70 s; education status ranges from college degree to doctoral degree; and employment status ranges from homemaker, employed and retired. Further detail on the overall demographics of the subjects in the Smartphone-PD study can be found in Zhan et al. [6].

**Figure 2:**
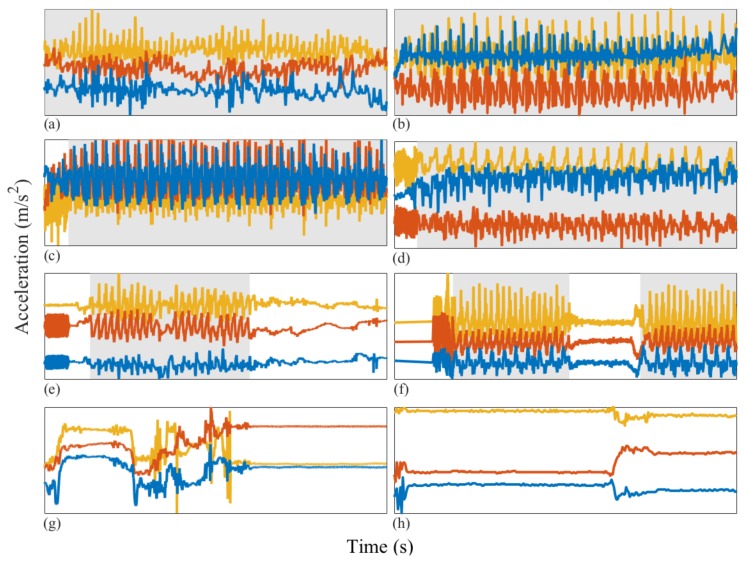
Illustrative examples of walking clinimetric test sensor data recorded using a smartphone accelerometer from healthy individuals and those with PD. Left column: walking tests from PD patients, right column: healthy individuals. The horizontal axis represents time in seconds (approximately 30 s) and the vertical axis (orange, red and blue) three-axis acceleration (m/s${}^{2}$). Grey shaded areas: data segments in which the user is hand-labeled as adhering to the test protocol. (**a**) PD patient walking throughout the test; (**b**) healthy individual walking throughout the test; (**c**) smartphone buzzer inn the first few seconds of the test, PD patient walking throughout the test; (**d**) buzzer recorded in the first few seconds of the test, healthy individual walking throughout the test; (**e**) buzzer recorded in the first few seconds of the test; the PD patient deviates from the test protocol before adhering to the test instructions by starting to walk, near the end of the test; the PD patient deviates from the test protocol; (**f**) buzzer captured in the first few seconds of the test; the healthy individual then begins walking, after which the individual deviates from the test protocol and resumes walking near the end of the test; (**g**,**h**) the PD patient and healthy individual both deviate from the test instructions throughout the test.

**Figure 3:**
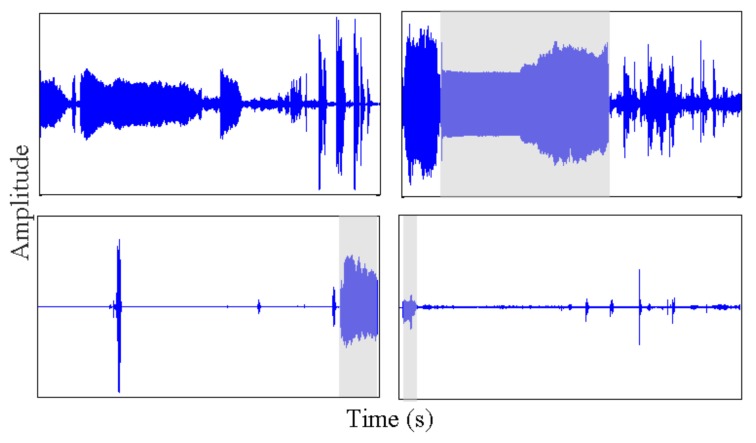
Illustrative examples of voice clinimetric test sensor data recorded using a smartphone microphone from healthy individuals and those with PD. The top two tests are performed by healthy individuals and the bottom two by PD patients. Voice tests take approximately 20 s, and the shaded area marks the part of the test where the user adheres to the test protocol.

In Figure 2, we present some illustrative examples of applying our hand-labeling protocol to walking clinimetric test sensor data collected from individuals with PD and healthy individuals. Similar illustration of the voice tests can be found in Figure 3.

### 2.3. Sensor-Specific Preprocessing

Whenever we analyse data from sensors, it is often necessary to apply some sensor-specific processing steps to the raw data to remove various confounders. This is the case for the walking, balance and voice tests described above.

#### 2.3.1. Isolating and Removing Orientation Changes from Accelerometry Data

One of the primary functions for which MEMs accelerometers were included in smartphones is to detect the orientation in which the user is holding the device and allow for the appropriate shift of the display between “landscape” (horizontal) and “portrait” (vertical) display modes. The accelerometer does this by measuring the Earth’s gravitational field acting on the smartphone. In clinimetric testing, accelerometers could be used to assess the ability of the user to perform certain daily activities that can be a strong indicator of a particular health condition. For example, it has been shown that PD can significantly affect activities such as walking or standing upright. In order to use the accelerometer data collected from a smartphone for monitoring walking (or balance), we first need to remove the effect of the Earth’s gravitational field from the raw accelerometer data, as it is a confounding factor.

Let us denote the raw accelerometer output that reflects the total acceleration due to forces applied to the device by $a_{r}\in R^{3}$; then, we can write:

(1) $$a_{r}=a_{d}+a_{g}$$

where $a_{g}\in R^{3}$ is the gravitational acceleration acting on the device and $a_{d}$ is the sum of the residual accelerations acting on the device (often called “linear” or “dynamic”) acceleration. We are interested in estimating $a_{d}$ from $a_{r}$ without observing $a_{g}$ directly. Without additional information, we have to make fairly strong assumptions about $a_{g}$ and $a_{d}$ in order to infer them. A common assumption is that orientation is locally stationary in time, so that passing the raw data through a digital high-pass filter of some sort is (under certain mathematical assumptions) the optimal solution. However, this assumption is too restrictive when the user is constantly interacting with the device and performing activity tests at the same time. At the same time, it is reasonable to assume that the measured gravitational field will follow relatively simple dynamics compared to the dynamic component. In this work, we propose a novel approach that models the gravitational field as a piecewise linear signal. This assumption is less restrictive than standard stationarity assumptions, but still allows us to rapidly filter away the effect of device orientation.

To estimate the unknown piecewise linear trend $a_{g}$ from the raw output $a_{r}$, we use $L_{1}$-trend filtering, which is a variation of the widely-used Hodrick–Prescott (H-P) filter [42]. The $L_{1}$-trend filter substitutes a sum of absolute values (i.e., an $L_{1}$ norm) for the sum of squares used in H-P filtering to penalise variations in the estimated trend. This is the second term in Equation (2), the effect of which is weighted by the filter hyperparameter λ.

**Figure 4:**
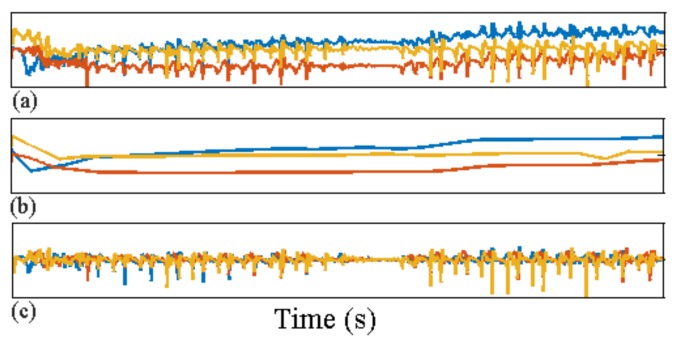
Raw accelerometer sensor output vector *x* for a single walking test (top panel); (**a**) raw acceleration data; (**b**) estimated gravitational orientation trend $\hat{g}$; (**c**) estimated dynamic acceleration after removing the effect of device orientation.

Assume we have *T* measurements of the raw accelerometer data (*T* data points), and let us denote them by $x_{1},\ldots ,x_{T}$ where $x_{t}\in R^{3}$ for t=1,…,T. The trend vectors $g_{1},\ldots ,g_{T}$ should minimise the objective function:

(2) $$\hat{g}=\arg\min_{g}\left\{\frac{1}{2}\sum_{t=1}^{T}{\left(x_{t}-g_{t}\right)}^{2}+\lambda \sum_{t=2}^{T-1}\left|g_{t-1}-2g_{t}+g_{t+1}\right|\right\}$$

where $\hat{g}={\hat{g}}_{1},\ldots ,{\hat{g}}_{T}$ denotes the set of gravitational vectors minimising the functional in (2), which defines the $L_{1}$-trend filter. The linear acceleration is then estimated by subtracting the estimated gravitational trends from the raw sensor output: $x_{t}^{d}=x_{t}-{\hat{g}}_{t}$ for t=1,…,T; see Figure 4.

#### 2.3.2. Feature Extraction

In contrast to existing algorithms for segmentation of sensor data, we propose a simple approach that can use just a single feature for each kind of sensor. The system could be easily extended to include a more sophisticated feature engineering stage; however, the benefits of this will depend on the clinimetric tests analysed.

We compute the magnitude of the three-axis dynamic acceleration vector estimated after preprocessing to remove the gravitational orientation component. If we denote the dynamic acceleration at time *t* by $x_{t}\in R^{3}$, then the magnitude is the Euclidean norm, and this is proportional to the magnitude of the instantaneous dynamic force being applied to the device (the missing constant of proportionality here is the combined, but unknown, mass of the device and the wearer). For quality control of both the walking and balance tests, we do not directly model the dynamic acceleration vector, only its magnitude (for the balance tests) and $\log_{10}$ magnitude (for the walking tests).

In order to efficiently process the data from the voice test, we also extract a single feature from the raw sensor output. The raw voice data used in this study are sampled at 44,100 Hz, and direct segmentation of this very high-rate signal would be an unnecessary computational challenge. Instead, we segment the original signal into 10-ms windows (as a common choice for the frame size in speech processing) and extract the signal energy of the data from each of the windows that contain 441 unidimensional (and dimensionless) sensor measurements.

#### 2.3.3. Down-Sampling

While we are interested in processing the data at sufficiently high frequency, in some situations, modeling the raw directly can be computationally wasteful when our interest is quality control only. We have studied the power spectrum of the different tests to find when we can down-sample the original high frequency signal to a lower frequency without losing essential information. In practice, appropriate down-sampling is particularly important whenever we use AR models. This is because high frequency data require inferring a high number of AR coefficients to accurately capture the dynamics of the data. Estimating large numbers of AR coefficients is difficult because parameter inference in the model requires high computational effort, and since the amount of data are always limited, it is also more likely to lead to unreliable estimates of the AR model parameters.

**Figure 5:**
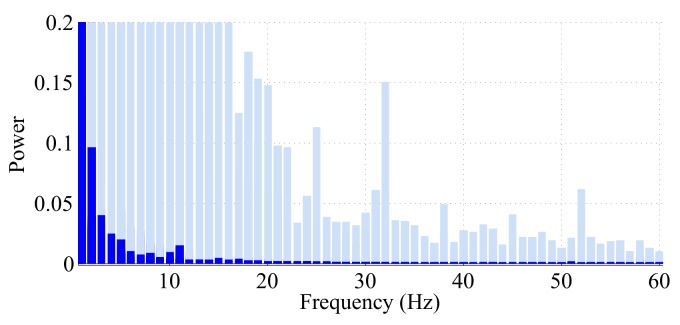
Power spectra of the magnitude of the three-axis accelerometer data each of the 100 balance and walking tests. The 200 densities are plotted on top of each other in order to see the maximum support that was observed at each frequency over all 200 tests. The shaded bars display the power spectra from the balance tests, and the colored bars denote the spectra of the walking tests. Since walking is highly periodic, in the spectra of these tests, most of the power is found in the lower frequencies associated with periodic walking. During the balance tests, periodic activities are observed for very short time durations, and the only periodic activity consistently recorded is the smartphone buzzer. This explains why we see that a lot of the power in the spectra is not found at the lower frequencies, but instead spread across the higher frequencies.

If we assume that the high frequency data recorded by the sensors consists of samples from a real signal *f*, then if *f* is band-limited, according to the Nyquist criterion, we can sample the original data at a rate near 2B (B>0 bandwidth of the signal) and reconstruct *f* perfectly from the down-sampled data. However, in the real world, most signals are not exactly band-limited, but their power spectrum shows a small magnitude at high frequencies; thus, we can apply a low pass filter to the original signal to make it band-limited. We evaluated the power spectrum of the accelerometer data from each of the 100 walking tests and the 100 balance tests. In Figure 5, we have combined all 100 densities for the walking tests and all 100 densities for the balance tests in the same plot. Figure 5 suggests strong evidence that the $\log_{10}$ magnitude of the linear acceleration from the walking tests comes from a nearly band-limited signal with band limit B<15 Hz. This observation is in agreement with the findings of [43] about the frequency content of walking data. Therefore, after removing the effect of the gravitational component, all the data coming from the walking tests are preprocessed with a low-pass filter of a cut-off frequency of 15 Hz. Then, from the Nyquist criterion, we can down-sample the signal to a uniform sampling rate of 2×15=30 Hz without risking aliasing.

There is little evidence (see Figure 5) to support the interpretation that the sensor data for the balance tests are band-limited; therefore, we omit the down-sampling step and model the magnitude of the accelerometer data from the balance tests in its original, high sample rate form.

### 2.4. Sequential Behavior Modeling

It is realistic to assume that for most remote health monitoring technology, detailed user behavior information would never be available after deployment. Therefore, traditional supervised machine learning activity recognition systems are not applicable, and we turn to unsupervised learning. We propose two different methods for segmenting distinct behaviors.

The first method is based on fitting a GMM to the data generated from each clinimetric test. The method does not require any labeled data for training, but imposes the strong assumption that the data violating the test protocols can be clustered into a different Gaussian component to data that adhere to the protocol. Despite the simplicity of this method, we demonstrate that in some scenarios, it manages to segment out most of the bad quality data points with very little computation involved.

For more complex scenarios, we also propose a general technique that can be used to segment different behaviors based on the properties of the data into some estimated number of different “states”.

#### 2.4.1. Unsupervised Behavior Modeling

**Figure 6:**
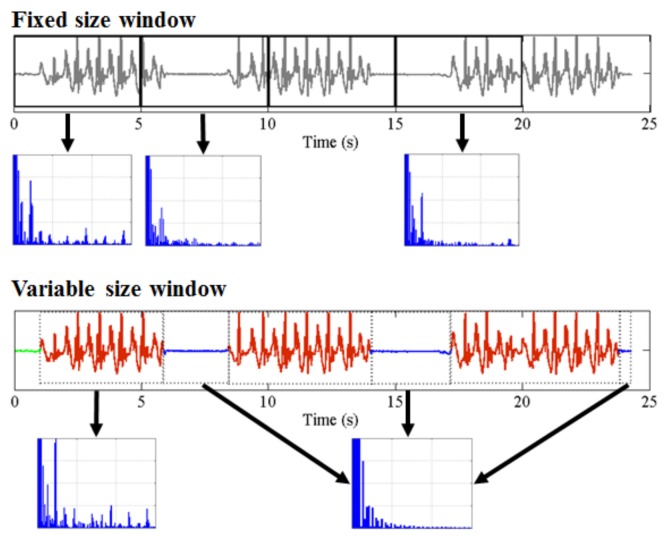
Demonstration of how the traditional feature extraction approach relying on windowing with fixed window size can lead to misleading estimates of any features from the frequency domain. The bottom plot demonstrates how an accelerometer signal is segmented using a switching AR model, and more accurate estimates of frequency domain features can be obtained as a result. We observe this by looking at how the power spectra change.

One of the primary methodological contributions of this work is obviating the need for elaborate feature engineering. Existing machine learning methods for activity recognition and segmentation of sensor data typically involve windowing the data at the start of the analysis and extracting a rich set of features from each data window with a prespecified fixed length (such as 30 s, 1 min, etc.). When it comes to the problem of quality control, in particular for clinimetric data, there are two major problems with this existing approach:

1. Any behavior changes that occur in the data within a window cannot be represented (see Figure 6). Due to our inability to model and account for them, they confound the feature values for the window in which they occur. Many of the features used for processing sensor data are some type of frequency domain feature (for example, dominant frequency component; largest magnitude Fourier coefficients; various wavelet coefficients, etc.). Frequency domain features are only meaningful for signals that have no abrupt changes; Fourier analysis over windows that contain abrupt discontinuities is dominated by unavoidable Gibbs’ phenomena [44]. Unfortunately, behavioral data from clinimetric tests are rife with such discontinuities due to inevitable changes in activities during tests. In the approach we propose, the window sizes and boundaries adapt to the data, since segmentation is learned using a probabilistic model that is specifically designed to capture rapid changes in activity when they occur, but also to model the intricacies of each activity.
2. The optimal features to be extracted from each window depend largely on the task/activity occurring in that window. If we are interested in developing a unified framework that works under a realistically wide set of scenarios encountered outside the lab, hand-picking an appropriate set of features for each activity that a clinimetric test might include is not feasible. This issue could be partially overcome if we use “automated” features such as principle component analysis (PCA), but this entails unrealistic assumptions (i.e., linearity). Alternatively, we could use an unsupervised approach for automated feature learning such as layers of restricted Boltzmann machines (RBM) or deep belief networks (DBN). However, these methods require large volumes of data from every behavior (which is unlikely to ever be available from health-impaired users), and sufficient computational power to train, making them unsuitable for deployment in real-time applications on smartphones or other resource-constrained devices. Even so, basic RBMs and DBNs would still need to be trained on features extracted after windowing of the sensor data. Additionally, although the features extracted using deep learning systems have demonstrated highly accurate classification results for many applications, we lack any ability to interpret these models to give a human understanding of what aspects of the data they represent. When dealing with healthcare applications, this lack of interpretability could significantly reduce the explanatory power required to gain confidence in the technique. The system we propose does not rely on extensive feature engineering or inscrutable deep learning algorithms, since we demonstrate sufficiently high performance using a single feature for each of the different sensor types. Of course, the proposed approach can be easily extended to use multiple features per data type, and this could potentially boost performance when appropriate features are chosen.

#### 2.4.2. Segmentation with GMMs

The simpler proposal is a GMM-based approach that relies on the assumption that (at least most of the time) the magnitude (for the accelerometry data, this is the length of the 3D acceleration vector, and for the voice data, this is the spectral power of each frame) of the sensor data adhering to the test protocols is different from the magnitude of the data violating them; or that we can approximately cluster the magnitude of that data into two separate Gaussian components. After we have applied the appropriate preprocessing depending on the data source (as described above), we fit a two-component (K=2) GMM to each of the different sensor datasets. The GMMs are estimated in an unsupervised way using the expectation-maximization (E-M) algorithm where after convergence, points are clustered to their most likely component using the maximum a posteriori (MAP) principle (more precisely, each point in time is assigned to the component that maximises the probability of its component indicator). Let us denote the preprocessed data by $x_{1},\ldots ,x_{T}$ with *T* being the number of sensor outputs for a given test after preprocessing. By fitting a K=2 component GMM to $x_{1},\ldots ,x_{T}$, we will estimate some indicators $z_{1},\ldots ,z_{T}$ that denote the component assignment of each time point (for example, $z_{t}=1$ denotes that time point $x_{t}$ is associated with Component 1). We denote by $\left(\mu_{1},\sigma_{1}\right)$ and $\left(\mu_{2},\sigma_{2}\right)$ the component mean and variance for the first and second component respectively. We use the estimated means $\mu_{1}$ and $\mu_{2}$ to identify whether the component corresponds to protocol adherence or violation. For walking tests and voice tests, we assume that if $\mu_{1}>\mu_{2}$, all data points $\left\{x_{t}:\;z_{t}=1\right\}$ represent adherence to the test protocols; hence, $\left\{x_{t}:\;z_{t}=2\right\}$ represent protocol violation. By contrast, for the balance tests, we assume that time points associated with the larger mean represent violation and the points associated with the smaller mean represent adherence to the protocols. This is because adherence in the walking tests results in higher acceleration, and adherence in the balance tests results in lower acceleration. Since the GMM ignores the sequential nature of the data (see Figure S1a, Supplementary Material), the estimated indicators $z_{1},\ldots ,z_{T}$ can switch very rapidly between the two components, providing an unrealistic representation of human behavior. In order to partially address this issue, we apply moving median filtering [45] to the indicator $z_{1},\ldots ,z_{T}$ and run it repeatedly to convergence. In this way, we obtain a “smoothed” sequence ${\hat{u}}_{1},\ldots ,{\hat{u}}_{T}$ that we use as classification of whether each of $x_{1},\ldots ,x_{T}$ is adhering to, or violating, the relevant protocol; time point *t* is classified as adherence if ${\hat{u}}_{t}=1$ and violation if ${\hat{u}}_{t}=2$.

#### 2.4.3. Segmentation with the Switching AR Model

In order to extend the GMM to model long time-scale dependence in the data, we can turn to HMMs [46]. HMMs with Gaussian observations (or mixtures of Gaussian observations) have long dominated areas such as activity [47,48,49] and speech recognition [46,50,51]. However, simple HMMs fail to model any of the frequency domain features of the data and are therefore not flexible enough to describe the sensor data; instead, we need to use a more appropriate model.

The switching AR model is a flexible discrete latent variable model for sequential data, which has been widely used in many applications, including econometrics and signal processing [52,53,54,55]. Typically, some *K* number of different AR models are assumed a priori. An order *r* AR model is a random process that describes a sequence $x_{t}$ as a linear combination of previous values in the sequence and a stochastic term:

(3) $$x_{t}=\sum_{j=1}^{r}A_{j}x_{t-j}+e_{t}\;e_{t}∼N\left(0,\sigma^{2}\right)$$

where $A_{1},\ldots ,A_{r}$ are the AR coefficients and $e_{t}$ is a zero mean, Gaussian i.i.d. sequence (we can trivially extend the model such that $e_{t}∼N\left(\mu ,\sigma^{2}\right)$ for any real-valued μ). The order *r* of the AR model directly determines the number of “spikes” in its spectral density, meaning that *r* controls the complexity or amount of detail in the power spectrum of $x_{t}$ that can be represented.

In switching AR models, we assume that the data comprise an inhomogeneous stochastic process, and multiple different AR models are required to represent the dynamic structure of the series, i.e.,

(4) $$x_{t}=\sum_{j=1}^{r}A_{j}^{z_{t}}x_{t-j}+e_{t}^{z_{t}}\;e_{t}^{z_{t}}∼N\left(0,\sigma_{z_{t}}^{2}\right)$$

where $z_{t}\in \left\{1,\ldots ,K\right\}$ indicates the AR model associated with point *t*. The latent variables $z_{1},\ldots ,z_{T}$ describing the switching process are modeled with a Markov chain. Typically, K≪T, allowing us to cluster together data that are likely to be modeled with the same AR coefficients.

The switching AR model above is closely related to the HMM: as with the switching AR model, the HMM also assumes that data are associated with a sequence of hidden (latent) variables that follow a Markov process. However, in the case of HMMs, we assume that given the latent variables, the observed data are independent. In other words, the simplest HMM can be considered as a switching AR model where the order *r* of each AR model is zero with non-zero mean error term. Neither of the models discussed here are necessarily limited to Gaussian data, and there have been HMM extensions utilising: multinomial states for part-of-speech tagging [56], Laplace distributed states for passive infrared signals [48] or even neural network observational models for image and video processing [57].

The segmentation produced with any variant of the HMM is highly dependent on the choice of *K* (the number of hidden Markov states, i.e., distinct AR models). In the problem we study here, the number *K* would roughly correspond to the number of different behavioral patterns that occur during each of the clinimetric tests. However, it is not realistic to assume we can anticipate how many different behaviors can occur during each test. In fact, it is likely that as we collect data from more tests, new patterns will emerge, and *K* will need to change. This motivates us to seek a Bayesian nonparametric (BNP) approach to this segmentation problem: a BNP extension of the switching AR model described above, which will be able to accommodate an unknown and changing number $K^{+}$ of AR models.

The nonparametric switching AR model (first derived as a special case of nonparametric switching linear dynamical systems in Fox et al. [58]) is obtained by augmenting the transition matrix of the HMM underlying the switching AR with a hierarchical Dirichlet process (HDP) [59] prior. Effectively, the HMM component of the switching AR model is replaced with an infinite HMM [60]. The infinite HMM avoids fixing the number of states *K* in the Markov model; instead, it assumes that the number of HMM states of an unknown, and potentially large $K^{+}$, and depends on the amount of training data we have already seen. Whenever we are fitting an infinite HMM, we typically start by assigning the data into a single hidden state (or a small fixed number of states), and at each step with some probability, we increase the number of effective states at each inference pass through the signal. In this way, it is possible to infer the number of effective states in an infinite HMM as a random variable from the data. The parameters specifying how quickly the number of effective states grows are called local and global concentration hyperparameters: α denotes the local and γ the global concentration.

The local α controls how likely it is that new types of transitions occur between the effective states, or essentially how sparse is the HMM transition matrix. The global γ reflects how likely it is for a new effective state to arise, or how many rows the transition matrix has. Unlike the fixed *K* in standard parametric HMMs, the hyperparameters α and γ of the infinite HMM (or any of its extensions) can be tuned with standard model selection tools that compute how the value of the complete data likelihood changes as α and γ change. This allows us to model the behavioral patterns in the smartphone clinimetric tests in a completely unsupervised way. For a lengthier discussion and derivation of the infinite HMM and the nonparametric switching AR model, we refer the readers to [58,59,60].

#### 2.4.4. Segmentation Context Mapping

The switching AR model groups together intervals of the preprocessed data that have similar dynamics described by the same AR pattern, i.e., we group points $x_{t}$ according to their corresponding indicator values $z_{t}=k$ for $k\in \left\{1,\ldots ,K^{+}\right\}$. The generality of this principle allows us to apply the framework widely across different datasets generated from diverse clinimetric tests such as walking, balance or voice tests.

A trained expert can reasonably identify intervals of walking or balancing that adhere to the corresponding test protocols, while specific physical activities would be difficult to identify purely from the accelerometer output. A lack of behavior labels can challenge our understanding of the segmentation from the previous stage. This motivated the collection of additional controlled clinimetric tests to shed some light on the patterns we discover using the nonparametric switching AR model. The controlled smartphone tests have been performed by healthy controls. We collect 32 walking, 32 balance and 32 voice tests in which we vary the orientation and location of the phone during a simulated clinimetric test. During these tests, subjects are instructed to perform some of the most common behaviors that we observe during clinimetric tests performed outside the lab. Activities conducted during the tests include freezing of gait, walking, coughing, sustained phonation, keeping balance and several others. A human expert annotated the monitored behaviors with $b_{1},\ldots ,b_{T}$, which associate each data point with a behavioral label (i.e., $b_{t}=“\mathrm{walking}”$ means point $x_{t}$ was recorded during walking). In contrast to the clinimetric tests performed outside the lab, here we have relatively detailed information about what physical behavior was recorded in each segment of these controlled tests.

Since we have the “ground truth” labels *b* for the controlled clinimetric tests, we can be confident in the interpretation of the intervals estimated by the unsupervised learning approach. This allows us to better understand the different intervals inferred from data collected from outside the lab, when labels *b* are not available. Note that $b_{1},\ldots ,b_{T}$ are not used during the training of the nonparametric switching AR model, but only for validation. Furthermore, the distribution of the data from the actual clinimetric tests collected outside of the lab significantly departs from the distribution of the data of the controlled tests.

We assess the ability of the model to segment data consisting of different behaviors. This is done by associating each of the unique $K^{+}$ values that the indicators *z* can take with one of the behavioral labels occurring during a controlled test. For each $k\in \left\{1,\ldots ,K^{+}\right\}$, state *k* is assumed to model behavior $b_{k}$ with $b_{k}=\mathrm{mode}\left\{b_{t}:z_{t}=k\right\}$ being the most probable behavior during that state.

Using this simple mapping from the numerical indicators $z_{1},\ldots ,z_{T}$ to interpretable behaviors, we obtain estimated behavior indicators ${\hat{z}}_{1},\ldots ,{\hat{z}}_{T}$. Using the estimated behavior indicators ${\hat{z}}_{1},\ldots ,{\hat{z}}_{T}$ and the “ground truth” labels $b_{1},\ldots ,b_{T}$, we compute the following algorithm performance measures: balanced accuracy (BA), true positive (TP) and true negative (TN) rates for the segmentation approach in Table 1. For example, given behavior $b^{*}$, these metrics are computed using:

(5) $$\begin{array}{c} \mathrm{TP}=\frac{\sum_{t=1}^{T}1\left({\hat{z}}_{t}=b^{*}\cap b_{t}=b^{*}\right)}{\sum_{t=1}^{T}1\left({\hat{z}}_{t}=b^{*}\right)};\mathrm{TN}=\frac{\sum_{t=1}^{T}1\left({\hat{z}}_{t}\neq b^{*}\cap b_{t}\neq b^{*}\right)}{\sum_{t=1}^{T}1\left({\hat{z}}_{t}\neq b^{*}\right)};\\ \mathrm{BA}=\frac{\mathrm{TP}+\mathrm{TN}}{2} \end{array}$$

where $1\left(\cdot \right)$ denotes the indicator function, which is one if the logical condition is true, zero otherwise.

Outside the lab, we cannot always label physical behaviors with high confidence. Instead, we use binary labels $u_{1},\ldots ,u_{T}$, which take values $u_{t}=1$ if point $x_{t}$ adheres or $u_{1}=2$ if it violates the applicable test protocol (as described in Section 2.2). In order to classify a time point $x_{t}$ with respect to its adherence to the protocol, it is sufficient to simply classify the state assignment $z_{t}$ associated with that time point.

To automate this context mapping, we use a highly interpretable naive Bayes classifier. We train the classifier using the posterior probabilities (we noticed that we can obtain very similar accuracy using just the modal estimates of the indicators $z_{1},\ldots ,z_{T}$ as an input to the classifier, which takes substantially less computational effort compared to computing the full posterior distribution of the indicators) of the indicators $z_{1},\ldots ,z_{T}$ associated with the training data as inputs and the corresponding binary labels $u_{1},\ldots ,u_{T}$ as outputs. For a new test point $\tilde{x}$, we can then compute the vector of probabilities $P\left(\tilde{z}\left|x_{1},\ldots ,x_{T},\theta ,\pi \right.\right)$ given the switching AR parameters θ and π (properly denoted in Figure S1b, Supplementary Material) and rescale them appropriately to appear as integer frequencies; we will write these vectors of frequencies as $p_{\tilde{z}}=\left(p_{\tilde{z},1},\ldots ,p_{\tilde{z},K^{+}}\right)$. The multinomial naive Bayes classifier assumes the following probabilistic model:

(6) $$P\left(p_{\tilde{z}}\left|u_{1},\ldots ,u_{T},\tilde{u},p_{z_{1},\ldots ,z_{T}}\right.\right)=\frac{\left(\sum_{k=1}^{K^{+}}p_{k}\right)!}{\prod_{k=1}^{K^{+}}p_{k}!}\prod_{k=1}^{K^{+}}{\bar{\pi }}_{k,\tilde{u}}^{p_{k}}$$

where ${\bar{\pi }}_{k,\tilde{u}}$ denotes the training probability for attribute *k* given the observation is from class $\tilde{u}$. This model can be then reversed (via Bayes rule) to predict the class assignment $\hat{u}\in \left\{1,2\right\}$, for some unlabeled input $p_{\tilde{z}}$:

(7) $$\hat{u}=\underset{c\in \left\{1,2\right\}}{\mathrm{arg max}}\left[\log P\left(\tilde{u}=c\right)+\sum_{k=1}^{K+}p_{k}\log\left({\bar{\pi }}_{k,c}\right)\right]$$

with $P\left(\tilde{p}=c\right)$ enabling control over the prior probabilities for class adherence/violation of the protocols.

**Figure 7:**
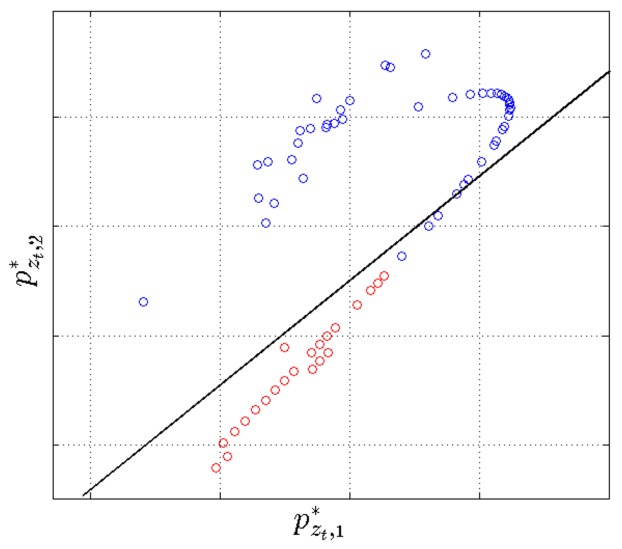
Visualising the switching AR posterior state probabilities for data from a single walking test. The log of the input state probabilities $p_{z_{1}},\ldots ,p_{z_{T}}$ is projected into 2D using linear discriminant analysis (LDA), and projections are denoted with $p_{z_{t}}^{*}$ where the $p_{z_{t}}$ is a $K^{+}$ dimensional vector and $p_{z_{t}}^{*}$ is 2D. Input projections are labeled with adherence (red) and violation (blue). The “linear” structure can be explained with the fact that typically, a data point $x_{t}$ is associated with high probability to only one of the $K^{+}$ AR states in the behavioral segmentation (and low probability for the remaining AR states) so that the input vector $p_{z_{t}}$ is sparse. The decision boundary of the multinomial naive Bayes classifier is also projected (using the same LDA coefficients) into 2D (black line). A few outlier projections $p_{z_{t}}^{*}$ are outside the plot axis limits, but they do not significantly affect the decision boundary and yet reduce the visual interpretability of the projection of the bulk of the data.

The multinomial naive Bayes is linear in the log-space of the input variables, making it very easy to understand; we demonstrate this by plotting a projection of the input variables and the decision boundary in 2D (Figure 7). The naive Bayes classifier requires very little training data to estimate parameters, scales linearly with the data size and despite its simplicity has shown performance close to state of the art for demanding applications such as topic modeling in natural language processing, spam detection in electronic communications and others [61]. One of the main disadvantages of this classifier is that it assumes, usually unrealistically, that the input variables are independent; however, this is not an issue in this application since the classifier is trained on a single feature. The multinomial naive Bayes classifier assumes that data in the different classification classes follow different multinomial distributions.

For different clinimetric tests, we need to train different classifiers because when the test protocols change, so does the association between the *z*’s and the *u*’s. However, the overall framework we use remains universal across the different tests and can be extrapolated to handle quality control in a wide set of clinimetric testing scenarios.

## 3. Results and Discussion

In order to evaluate the performance of the proposed framework, data from 300 clinimetric tests (100 walking, 100 balance and 100 voice tests) performed by PD patients and healthy controls from the Smartphone-PD study (see Zhan et al. [6]) were processed using the steps described above. The accelerometer data from the walking and balance tests are recorded at frequency rates varying between 50 Hz and 200 Hz. It is interpolated to a uniform rate of 120 Hz (using standard cubic spline interpolation), and the orientation is removed using $L_{1}$-trend filtering as described in Section 2.3.1. We extract the log-amplitude of the dynamic acceleration component for the walking tests and the amplitude of the dynamic component for the balance tests, which serve as input to the behavioral segmentation step. The log-amplitude for the walking tests is also down-sampled by a factor of four, resulting in a length of ~90,000 one-dimensional sequential preprocessed time series; balance tests are not down-sampled giving a length of ~241,000 one-dimensional time series. For the voice tests, the extracted energy of each 10-ms frame consists of a length of ~200,000 one-dimensional energy time series.

First, the two-component GMM-based approach described above is evaluated for each of the three datasets where performance is reported in Table 2. The metrics are estimated using the expressions (5) where we compare the estimated binary indicators ${\hat{u}}_{1},\ldots ,{\hat{u}}_{T}$ and “ground truth” labels $u_{1},\ldots ,u_{T}$ denoting adherence/violation. Since this approach is completely unsupervised, we use only the data for training and use the labels for validation only. While the GMM is not flexible enough to model the full composite behavioral complexity of the data captured during most clinimetric tests, we observe high accuracies for the voice tests. This is because the protocol for voice tests consist of producing sustained vocal phonations in very close proximity to the sensor. Therefore, it can be argued that adherence to the protocol for this activity is distinguishable based on an appropriate measure of magnitude of the sensor recordings alone, largely ignoring the longer-scale temporal variations. In clinimetric tests where the protocol requires the user to perform behaviors with more complex, composite dynamics, the limitations of the simple GMM become more apparent. For example, this occurs during the walking tests where protocol violations can be distinguished a lot more accurately if the behavioral segmentation model also incorporates both the longer-term sequential nature of the data and its spectral information.

Next, the nonparametric switching AR behavioral segmentation model is fitted to each of the three datasets where we specify the maximum order of the AR models associated with each state to r=4. For the evaluation, here parameter inference is performed using the truncated block Gibbs sampler described in Fox et al. [58]. For future, real-time deployment, we can use more scalable deterministic algorithms based on extensions of Hughes et al. [62], Raykov et al. [63].

As described above, we now input the state indicators $z_{1},\ldots ,z_{T}$ to a multinomial, naive Bayes classifier where if an indicator value has not been seen during training, we assume it is classified as a violation of the protocol during monitoring. The ability of the method to correctly classify adherence to and violations of the protocols of the three tests is measured using standard 10-fold cross-validation. The mean and standard deviation of the BA, TP and TN rates of the classifier are shown in Table 2. Note that in contrast to Section 2.4.4, the accuracy metrics are now comparing the binary labels *u* and estimated $\hat{u}$. Both the TP and TN values are consistently high across all tests, but the fact that the TN values are close to 90% for all three tests suggests a low probability of incorrectly labeling data that adhere to the test protocol as a violation of the protocol. In practice, the confidence in this prediction of adherence/violation of the protocol can be assessed using the state assignment probabilities in the naive Bayes classifier associated with each time series data point (the state assignment probabilities for each class of the naive Bayes are the terms inside the arg max operator in (6) after normalisation).

In order to ensure that the reported classification accuracy is due to the meaningful segmentation produced by the nonparametric switching AR model, we also report the performance of a multinomial naive Bayes classifier (Table 2) trained on the shuffled state indicators estimated via the nonparametric switching AR model during the segmentation stage. In this way, the classifier is trained on identical data, but with randomised association between the data and the training labels (i.e., $\left\{z_{1},\ldots ,z_{T}\right\}$ are randomly permuted while keeping $\left\{u_{1},\ldots ,u_{T}\right\}$ fixed). If the association between estimated state indicators and training labels is an accurate representation, we would expect a classifier trained on the shuffled indicators to score a balanced accuracy of around 50%; see Table 2.

### 3.1. Future Work

#### 3.1.1. Simultaneous Multimodal Sensing

The proposed framework is not limited to specific types of clinimetric activity tests nor specific sensors. It was demonstrated that it can be applied to voice and accelerometer data from three different smartphone clinimetric tests, but the approach could be easily generalised to different sensor-generated time series. For example, it would be straightforward to preprocess multiple sensor types obtained simultaneously during a test and then model these combined sensors together to perform segmentation. This may increase the accuracy of quality control.

#### 3.1.2. Real-Time Deployment

Mobile, sensor-based applications that can support research into the detection and symptom monitoring of PD have already been deployed on a large scale outside the lab. Incorporating the proposed system into PD research apps such as mPower could provide automatic, real-time quality control of the smartphone clinimetric tests. This could automatically remove any data that violate the test protocol, eliminating the need to store, transfer or analyse unwanted data.

The most computationally-demanding component of the proposed system is the inference of the nonparametric switching AR model. However, the switching AR model can be seen as an extension of the infinite HMM. Therefore, in order to perform real-time inference of the model on smartphone or wearable devices, we can use the computational optimisation proposed in Raykov et al. [48] and Leech et al. [64], which enables the inference of an infinite HMM on a highly resource-constrained microcontroller.

#### 3.1.3. Contextual Learning

“Passive monitoring”, where sensor data are captured in an entirely ambulatory way under realistic conditions outside the lab, provides a way to study PD symptoms objectively without interrupting routine activities. The successful monitoring of such daily behavioral details may provide unprecedented insight into the objective monitoring of individuals living with PD. However, outside the lab, we usually have little information about the routine activities under measurement unless other, simultaneous monitoring methods are used, such as video recording. However, video monitoring of patients in their homes is expensive and can impact the integrity of the data as it is highly invasive; patient behavior may be altered under the awareness of video monitoring. In addition, without multiple cameras in each room, it is not possible to follow patients at different locations, which means that videoing every daily activity a patient performs is impractical. The system proposed here can be trained to recognise specific patient activities and help researchers identify segments of the passive monitoring data that are most relevant for subsequent analysis. For example, consider patients being passively monitored using smartphones, where researchers wish to assess the effect of some medication on symptoms such as slowness of movement [65] or postural sway [7]. With very few labeled instances of the relevant behaviors, the system proposed here can learn to identify walking and balance behaviors from the continuous, passive sensor data, which will assist researchers into objectively testing their hypothesis.

### 3.2. Limitations

Due to the largely unsupervised nature of the proposed approach, it can be very easily extended to a variety of clinimetric tests with varying test protocols. However, this also means that in the segmentation step, we are unlikely to locate clusters of composite human behaviors. That is, in the example application presented here, the smartphone test protocols consisted of simple physical activities such as walking and pronunciation of a sustained phonation. However, more complex human activities can include a combination of simple activities: cooking includes components of walking and multiple hand activities; different sports can include a combination of walking, running and other simple activities, etc. This means that clinimetric tests with more complex protocols are likely to introduce additional segmentation challenges that might require a larger amount of labeled data and a more sophisticated segmentation model.

## 4. Conclusions

In this paper, we have introduced an end to end framework for quality control of sensor data collected during clinimetric tests for remote patient monitoring outside the lab. We have proposed two different methods for segmenting ‘good’ quality and ‘bad’ quality data: the first is based on GMMs, and the second is based on a nonparametric switching AR model. Both methods bypass the need for dubious feature engineering, but explore different trade-offs between activity segmentation accuracy and computational efficiency. The simpler GMM approach assumes that the data associated with adherence to clinimetric protocols mostly has a higher magnitude compared to data associated with violation of the protocols. By contrast, the more flexible semi-supervised nonparametric switching AR model is capable of inferring structural differences in sensor data recorded during different behaviors; the learned parametrisation of the different behaviors can then be used to map which behaviors adhere to or violate clinimetric test protocols. The quality control system developed here achieves accuracies of up to 90% across different clinimetric tests and different types of sensor data. With minimal effort, it can be used to clean and analyse in a more interpretable way data generated from multiple digital health applications.

## Figures and Tables

**Table 1:** Balanced accuracy (BA), true positive (TP) and true negative (TN) rates for the nonparametric switching AR model trained on walking and balance tests performed in a controlled environment. The TP rate reflects the ability to correctly identify an activity when occurring, and the TN rate reflects the ability to correctly indicate the lack of that activity whenever it is not occurring.

Behavior	BA	TP	TN
Walking	95%	96%	93%
Standing up straight	95%	98%	91%
Phone stationary	98%	100%	95%
Sustained phonation	98%	99%	97%

**Table 2:** Performance of the two proposed algorithms for quality control of clinimetric data and comparison with the randomised classifier. For the naive Bayes and the randomised classifiers, quality control predictions over all time points are evaluated using 10-fold cross-validation. We report the mean and standard deviation (in the brackets) of the balanced accuracy (BA), true positive (TP) and true negative (TN) rates across the different cross-validation trials changing the subsets of data used for training and testing. For the GMM-based approach, we report the BA, TP and TN rates using all the data for training and for testing since this approach is completely unsupervised; the standard deviation is not meaningful for a single trial (hence, standard deviations are omitted).

	Walking Tests	Balance Tests	Voice Tests
Nonparametric switching AR + naive Bayes			
BA	85% (11%)	81% (14%)	89% (8%)
TP	85% (18%)	81% (16%)	88% (9%)
TN	90% (8%)	88% (9%)	91% (9%)
GMM + running median filtering			
BA	62%	24%	99%
TP	80%	74%	86%
TN	89%	82%	96%
randomised classifier			
BA	50% (1%)	50% (0.2%)	53% (24%)
TP	1% (0.4%)	0.4% (0.02%)	99% (1%)
TN	100%	100%	6% (23%)

## Supplementary Materials

The following are available online at http://www.mdpi.com/1424-8220/18/4/1215/s1.
