# Supplementary material for: Automated Quality Control for Sensor Based Symptom Measurement Performed Outside the Lab
Source: Sensors (Basel). 2018 Apr 16;18(4):1215. doi: 10.3390/s18041215 (PMC5948536; doi:10.3390/s18041215)
Supplement: Supplementary file 1 [file sensors-18-01215-s001.pdf]

Supplementary Material

Table S1:
Clinimetric test protocols, assumptions and commonly encountered protocol violations in the Smartphone-PD project data used to test the quality control framework proposed in this study.

Test	Protocol	Protocol violations	Hand-labelling protocol
Voice	Place the phone up to your ear as if making a normal phone call. Take a deep breath, and say "aaah" for as long as you can, at a steady volume and pitch.	1. User interactions with the smartphone including taking a phone call, texting or playing a game during test.<br>2. User performs test in loud environment.<br>3. Non-sustained vowel phonation activities including coughing, reading out the instructions on the display, talking to another person, during test.	(1) Vowel sound segments are marked as adherence<br>(2) Anything else is marked as non-adherence
Balance	Place the phone in your pocket. When the buzzer vibrates, stand up straight unaided.	1. User interactions with the smartphone including taking a phone call, texting or playing a game during test.<br>2. User is jumping or falling during the test.	(1) Where there is uncertainty in the user's activity, a non-adherence label is applied<br>(2) Buzzer is labelled as non-adherence<br>(3) Where an interval is confounded with the buzzer, a non-adherence label is given to that interval<br>(4) Where an interval is confounded with an orientation change of the smartphone and the data is otherwise ambiguous, a non-adherence label is given to that interval
Walking	Stand up and place the phone in your pocket. When the buzzer vibrates, walk forward 20 yards; then turn around and walk back.	1. User interactions with the smartphone including taking a phone call, texting or playing a game during test.<br>2. Non-walking activities including jumping, falling or standing still.<br>3. User encounters obstacles during walking which interfere with normal walking.	(1) Where there is uncertainty in the user's activity, a non-adherence label is applied<br>(2) Buzzer is labelled as non-adherence<br>(3) Where an interval is confounded with the buzzer, a non-adherence label is given to that interval<br>(4) Where an interval is confounded with an orientation change of the smartphone and the data is otherwise ambiguous, a non-adherence label is given to that interval<br>(5) A turn is labelled as adherence

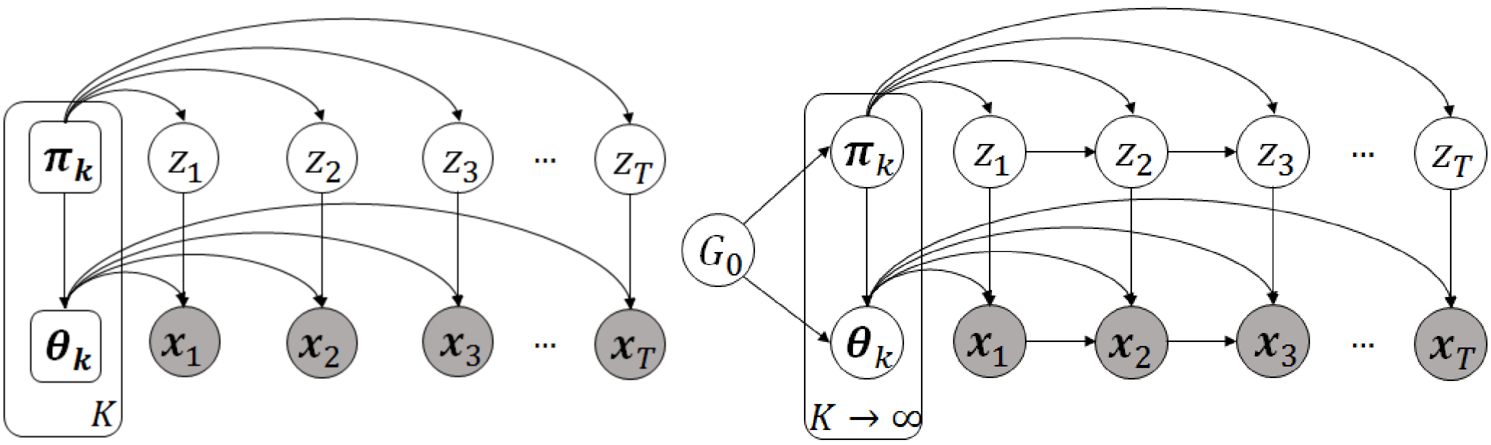

(a) (b)

Figure S1: Probabilistic graphical models for (a) Gaussian mixture model (GMM) and (b) nonparametric switching autoregressive (AR) model. The GMM component parameters θ_k are the mean and the variance of data associated with component k. By contrast, the component parameters θ_k for the switching AR consist of the AR coefficients $A_1^k, \ldots, A_r^k$ and the AR error parameters μ_k and σ_k. Parameter π_k denotes the mixing coefficients and the transition matrix for the GMM and the switching AR, respectively. In the parametric GMM π_k and θ_k are fixed. In the nonparametric switching AR π_k and θ_k are modeled with an HDP prior, where $G = \{G_1, \ldots, G_{K+}\}$ $\sim$HDP$(\alpha, \gamma, \theta_0)$. Hyperparameter θ_0 denotes the conjugate prior over the $A's$, the $\mu's$ and the $\sigma's$. See main text for further description of the HDP and its concentration parameters α and γ.
